# The impact of serine protease HtrA in apoptosis, intestinal immune responses and extra-intestinal histopathology during *Campylobacter jejuni* infection of infant mice

**DOI:** 10.1186/1757-4749-6-16

**Published:** 2014-05-27

**Authors:** Markus M Heimesaat, André Fischer, Marie Alutis, Ursula Grundmann, Manja Boehm, Nicole Tegtmeyer, Ulf B Göbel, Anja A Kühl, Stefan Bereswill, Steffen Backert

**Affiliations:** 1 Department of Microbiology and Hygiene, Charité - University Medicine Berlin, Campus Benjamin Franklin, Hindenburgdamm 27, D-12203 Berlin, Germany; 2 Department of Biology, Division of Microbiology, Friedrich Alexander University Erlangen, Nuremberg, Germany; 3 Department of Medicine I for Gastroenterology, Infectious Disease and Rheumatology / Research Center ImmunoSciences (RCIS), Charité - University Medicine Berlin, Berlin, Germany

**Keywords:** Cell invasion, Conventional infant mice, Ulcerative enterocolitis, Innate immunity, Host-pathogen-interaction, Apoptosis, Extra-intestinal immune responses, Hepatic, Renal, Pulmonal histopathology

## Abstract

**Background:**

*Campylobacter jejuni* has emerged as a leading cause of bacterial enterocolitis. The serine protease HtrA has been shown to be a pivotal, novel *C. jejuni* virulence factor involved in cell invasion and transmigration across polarised epithelial cells *in vitro*. However, the functional relevance of the *htrA* gene for the interaction of *C. jejuni* with the host immune system in the infant mouse infection model has not been investigated so far.

**Results:**

Here we studied the role of *C. jejuni htrA* during infection of 3-weeks-old infant mice. Immediately after weaning, conventional wild-type mice were perorally infected with the NCTC11168∆*htrA* mutant (∆*htrA*) or the parental wild-type strain. Approximately one third of infected infant mice suffered from bloody diarrhea until day 7 post infection (p.i.), whereas colonic histopathological changes were rather moderate but comparable between the two strains. Interestingly, parental, but not ∆*htrA* mutant infected mice, displayed a multifold increase of apoptotic cells in the colonic mucosa at day 7 p.i., which was paralleled by higher colonic levels of pro-inflammatory cytokines such as TNF-α and IFN-γ and the matrix-degrading enzyme matrixmetalloproteinase-2 (MMP-2). Furthermore, higher numbers of proliferating cells could be observed in the colon of ∆*htrA* infected mice as compared to the parental wild-type strain. Remarkably, as early as 7 days p.i. infant mice also exhibited inflammatory changes in extra-intestinal compartments such as liver, kidneys and lungs, which were less distinct in kidneys and lungs following ∆*htrA* versus parental strain infection. However, live *C. jejuni* bacteria could not be found in these organs, suggesting the induction of systemic effects during intestinal infection.

**Conclusion:**

Upon *C. jejuni* ∆*htrA* strain infection of infant mice, intestinal and extra-intestinal pro-inflammatory immune responses were ameliorated in the infant mouse model system. Future studies will shed further light onto the molecular mechanisms of host-pathogen interactions.

## Background

*Camplylobacter jejuni* displays a major infectious agent of foodborne bacterial enterocolitis of men with increasing prevalence in developed as well as developing countries [1,2]. Severity of campylobacteriosis varies from mild disease to acute symptoms such as abdominal cramps, fever, myalgia, and watery to bloody diarrhea [3]. Patients suffering from acute disease display crypt abscesses, ulcerations and colonic infiltration with pro-inflammatory immune cell populations [4-6]. Whereas the vast majority of *C. jejuni* infections is normally self-limiting in humans, post-infectious sequelae such as Guillain-Barré syndrome, Miller Fisher syndrome, Reiter’s syndrome and reactive polyarthritis might arise in rare cases [3,7]. An important prerequisite for *C. jejuni* causing disease is its ability to adhere and invade intestinal epithelial cells [8]. A plethora of bacterial outer membrane proteins such as JlpA, CadF, FlpA, PEB1 among others has been shown to be involved in adhesion to epithelial cells [9-13], whereas CadF can induce the activation of small Rho GTPases, Rac1 and Cdc42, which exert invasive properties *in vitro*[13-16] and in human *ex vivo* biopsies [17]. We and others have recently shown that the *C. jejuni* serine protease and chaperone HtrA (high temperature requirement A) displays a novel virulence factor [18-21]. Whereas HtrA family members were considered in the past to strictly act intracellularly in the bacteria, we recently discovered that HtrA is actively secreted into the extracellular environment where it cleaves cell surface adhesion proteins and tumor-suppressor E-cadherin [21-23]. *In vitro* infection experiments with *C. jejuni* revealed that secreted HtrA is capable of opening cell-to-cell-junctions in the epithelium by cleaving-off the 90 kDa extracellular domain of E-cadherin [21,22]. Furthermore, *htrA* gene deletion has been shown to result in defective E-cadherin shedding and compromised transmigration of *C. jejuni* across polarized epithelial cells *in vitro*[21].

The studies of molecular mechanisms of pathogen-host-interactions causing *C. jejuni* induced disease have been hampered by a lack of suitable *in vivo* models given that the host-specific composition of the microbiota determines the physiological colonization resistance against *C. jejuni*[24,25]. Whereas conventionally colonized adult (>8-weeks-old) mice expel the pathogen within a few days post infection, gnotobiotic wild-type mice and mice recolonized with human microbiota were readily colonized by *C. jejuni*[24]. However, classical clinical symptoms of human campylobacteriosis such as bloody diarrhea were missing in these murine infection models [24]. In contrast 3-weeks-old infant mice are highly susceptible to *C. jejuni* infection and develop self-limiting bloody diarrhea within one week [25-30]. After resolving enterocolitis within another 7–10 days, infant mice were asymptomatic long-term *C. jejuni* carriers exhibiting distinct pro-inflammatory immune responses in intestinal as well as extra-intestinal locations such as liver, lungs, and kidneys characterized by influx of predominantly T (and less distinctly B) lymphocytes after more than 3 months p.i. [25,31]. In the present study, we applied the infant mouse model to investigate the functional relevance of the *htrA* gene in *C. jejuni* infection *in vivo*. Furthermore we studied potential extra-intestinal inflammatory sequelae in the early course of *C. jejuni* induced disease.

## Results

### Intestinal colonization and clinical symptoms in infant mice following infection with wild-type and *htrA* mutant *C. jejuni*

**Figure 1:**
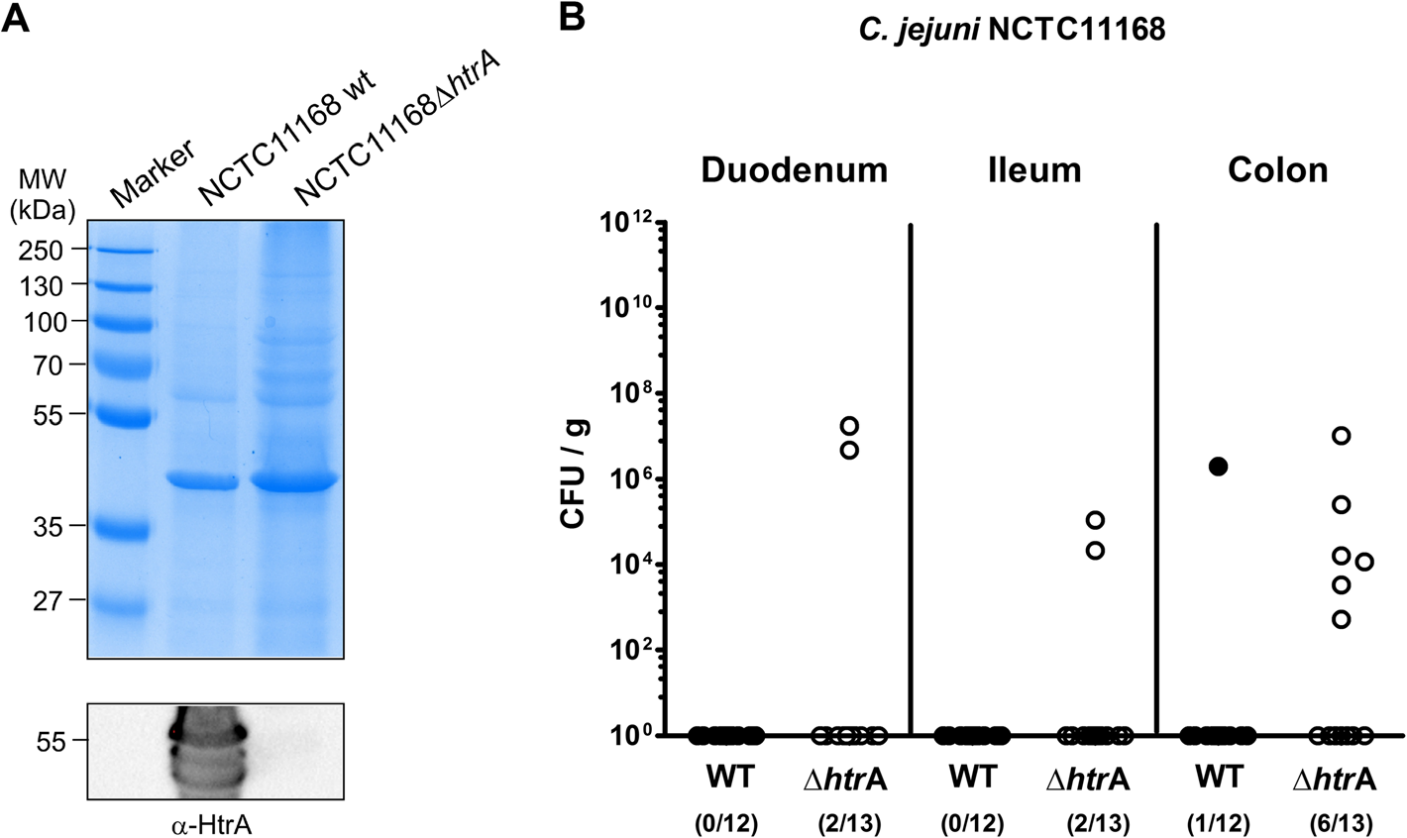
**Influence of HtrA on *C. jejuni *colonization along the intestinal tract of infant mice. (A)** Loading control (Coomassie blue staining) of proteins from total cell lysates (top) and Western blot for the expression of HtrA in the two *C. jejuni* strains used in this infection study. **(B)** Immediately after weaning, conventional infant wild-type mice were perorally infected with *C. jejuni* NCTC11168 (WT, closed circles) or the mutant strain NCTC11168∆*htrA* (∆*htrA*, open circles). The pathogenic loads in distinct compartments of the intestinal tract were determined by quantification of live *C. jejuni* in luminal samples taken from duodenum, ileum, and colon at day 7 following infection by cultural analysis (CFU, colony forming units). Medians (black bars) are indicated and numbers of mice harbouring the respective *C. jejuni* strain out of the total numbers of analyzed animals are given in parentheses. Data shown were pooled from three independent experiments.

**Figure 2:**
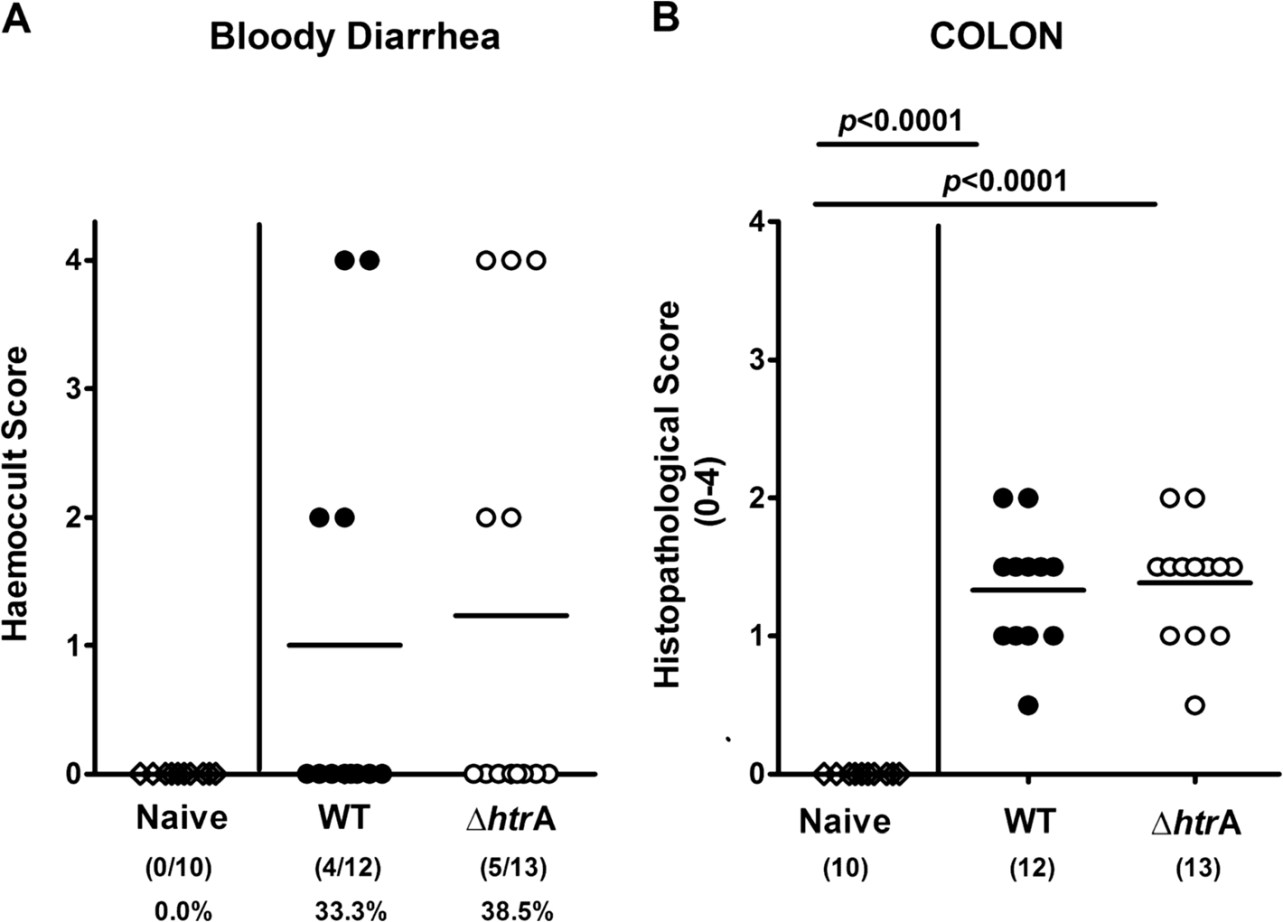
**Role of HtrA in clinical symptoms and colonic histopathology in *C. jejuni *infected infant mice.** Immediately after weaning, infant mice were perorally infected with *C. jejuni* NCTC11168 (WT, closed circles) or the mutant strain NCTC11168∆*htrA* (∆*htrA*, open circles). Uninfected animals (Naïve; open diamonds) served as negative controls. Seven days following *C. jejuni* infection **(A)** occurrence of blood in fecal samples and **(B)** histopathological changes in H&E stained colonic paraffin sections were assessed by applying standardized clinical and histophathological scoring systems, respectively. Means (black bars) and numbers of analyzed animals (in parentheses) or absolute and relative (in %) numbers of positive samples out of the total number are indicated. Data shown were pooled from three independent experiments.

Immediately after weaning conventional 3-weeks-old infant mice were perorally infected with approximately 10^9^ colony forming units (CFU) of either the *C. jejuni* knockout mutant NCTC11168::*htrA* (∆*htrA*) or the parental wild-type (WT) strain each harvested in the stationary phase on two consecutive days (day 0 and 1). Control analyses demonstrated that equal amounts of *C. jejuni* protein were infected per sample and the HtrA protein is not expressed in the Δ*htrA* mutant as expected (Figure 1A). Seven days post infection (p.i.) less than half of parental and ∆*htrA* strain infected mice harboured the pathogen in the large intestine (8.3% and 46.2%, respectively) with relatively low pathogenic loads between 10^3^ and 10^7^ CFU per g luminal content, whereas in the proximal and distal small intestinal tract either *C. jejuni* strain was virtually undetectable (Figure 1B). In addition, approximately one third of mice developed clinical symptoms of *C. jejuni* induced acute enterocolitis until day 7 p.i., as indicated by 33.3% and 38.5% positive cases of bloody diarrhea in parental and ∆*htrA* strain infected mice, respectively (Figure 2A). We next assessed gradual histopathological changes in the mucosa and lamina propria of hematoxylin and eosin (H&E) stained colonic paraffin sections. Seven days following infection with either strain mice displayed comparable histopathological scores and exhibited rather mild to moderate colonic histopathological changes such as single to mild scattered cell infiltrates within the colonic mucosa and submucosa, mild epithelial hyperplasia and starting loss of goblet cells (Figure 2B).

### *C. jejuni* HtrA aggravates intestinal apoptosis and immune responses

**Figure 3:**
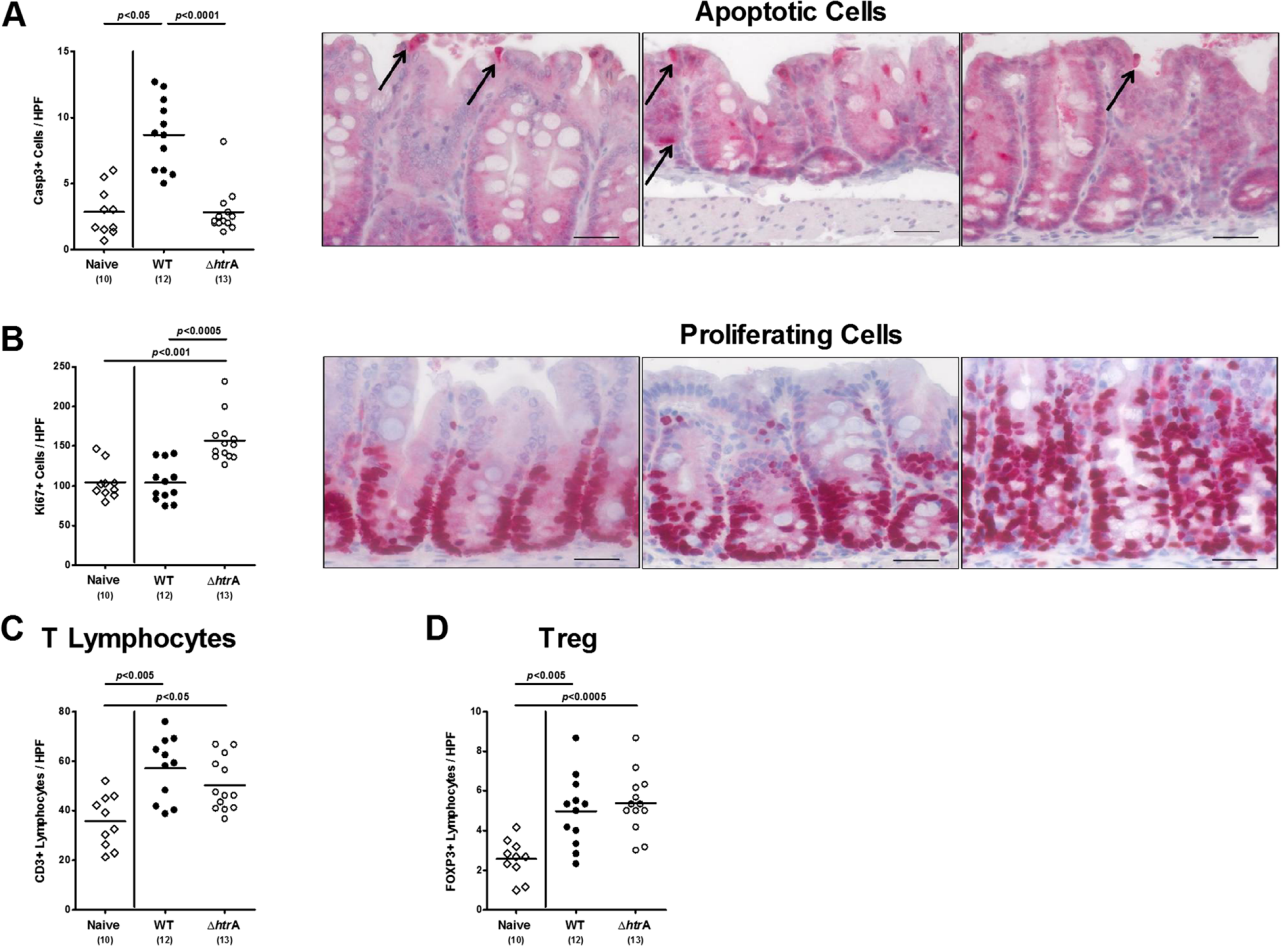
**HtrA aggravates *C. jejuni *induced colonic apoptosis and inflammation.** Immediately after weaning, infant mice were perorally infected with *C. jejuni* NCTC11168 (WT, closed circles) or the mutant strain NCTC11168∆*htrA* (∆*htrA,* open circles). Uninfected animals (Naïve; open diamonds) served as negative controls. The average numbers of apoptotic cells (positive for caspase-3, panel **A**), proliferating cells (positive for Ki67, panel **B**), T lymphocytes (positive for CD3, panel **C**), and regulatory T cells (Treg, positive for Foxp3; panel **D**) from at least six high power fields (HPF, 400× magnification) per animal were determined microscopically in immunohistochemically stained colonic sections at day 7 p.i. In addition, representative photomicrographs of colonic Casp3-positive apoptotic (indicated by black arrows; **A**, right panel) and Ki67-positive proliferating (**B**, right panel) cells are shown (Naïve left, WT middle, ∆*htrA* right photo; 400× magnification, scale bar 20 μm). Numbers of analyzed animals are given in parentheses. Means (black bars) and levels of significance (*P*-values) determined by the Mann–Whitney-U test are indicated. Data shown were pooled from three independent experiments.

Given that apoptosis is a commonly used diagnostic marker in the histopathological evaluation and grading of intestinal disease [24] and a key feature of *C. jejuni* induced ulcerative enterocolitis [25], we next quantitatively assessed apoptotic cells applying *in situ* immunohistochemical stainings of colonic paraffin sections. Seven days following parental, but not ∆*htrA* strain infection, infant mice displayed more than two fold higher colonic caspase3-positive apoptotic cell numbers as compared to naïve controls (p < 0.05 vs. naïve; p < 0.0001 vs. ∆*htrA* strain; Figure 3A). Furthermore, ∆*htrA* strain infected mice exhibited more colonic Ki67-positive proliferating cells as compared to parental strain infected and naïve controls (p < 0.0005 and p < 0.001, respectively; Figure 3B) indicative for upregulated regenerative epithelial function during immunopathology. Given that recruitment of immune cells is a hallmark of human campylobacteriosis [4,5] we next quantitatively assessed T cell populations in the large intestines of *C. jejuni* infected mice. Seven days following ∆*htrA* strain infection, infant mice displayed a trend towards lower colonic CD3-positive T lymphocyte numbers as compared to parental strain infected control animals (not significant due to high SD in either group; Figure 3C), whereas Foxp3-positive regulatory T cells (Tregs) increased comparably in the colonic mucosa and lamina propria of mice upon peroral infection with either strain (Figure 3D).

### *C. jejuni* HtrA is necessary for the induction of TNF-α, IFN-γ and matrixmetalloproteinase-2

**Figure 4:**
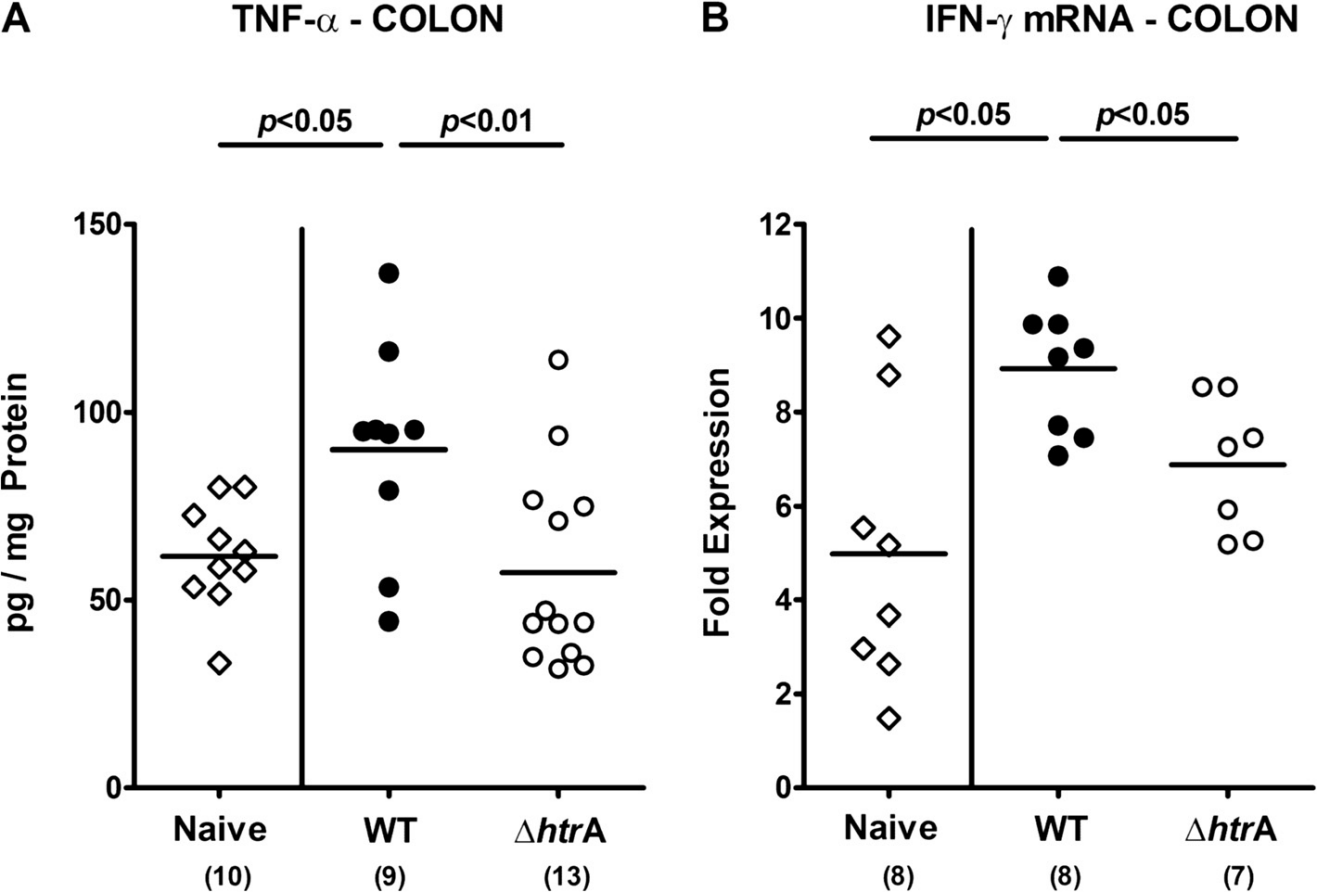
**HtrA impacts colonic pro-inflammatory cytokine expression during *C. jejuni *infection.** Immediately after weaning, infant mice were perorally infected with *C. jejuni* NCTC11168 (WT, closed circles) or the mutant strain NCTC11168∆*htrA* (∆*htrA*, open circles). Uninfected animals (Naïve; open diamonds) served as negative controls. **(A)** TNF-α protein and **(B)** IFN-γ mRNA expression levels were determined in *ex vivo* biopsies taken from colons at day 7 p.i. by ELISA and quantitative real time PCR technique, respectively. Numbers of analyzed animals are given in parentheses. Means (black bars) and levels of significance (*P*-values) determined by the Mann–Whitney-U test are indicated. Data shown were pooled from at least two independent experiments.

We next determined *C. jejuni* induced pro-inflammatory cytokine responses in colonic *ex vivo* biopsies. Colonic TNF-α protein and IFN-γ mRNA expression levels increased almost two-fold 7 days following parental, but not ∆*htrA* infection (p < 0.01 and p < 0.05, respectively; Figure 4) further underlining the role of HtrA in aggravating *C. jeuni* mediated inflammation.

**Figure 5:**
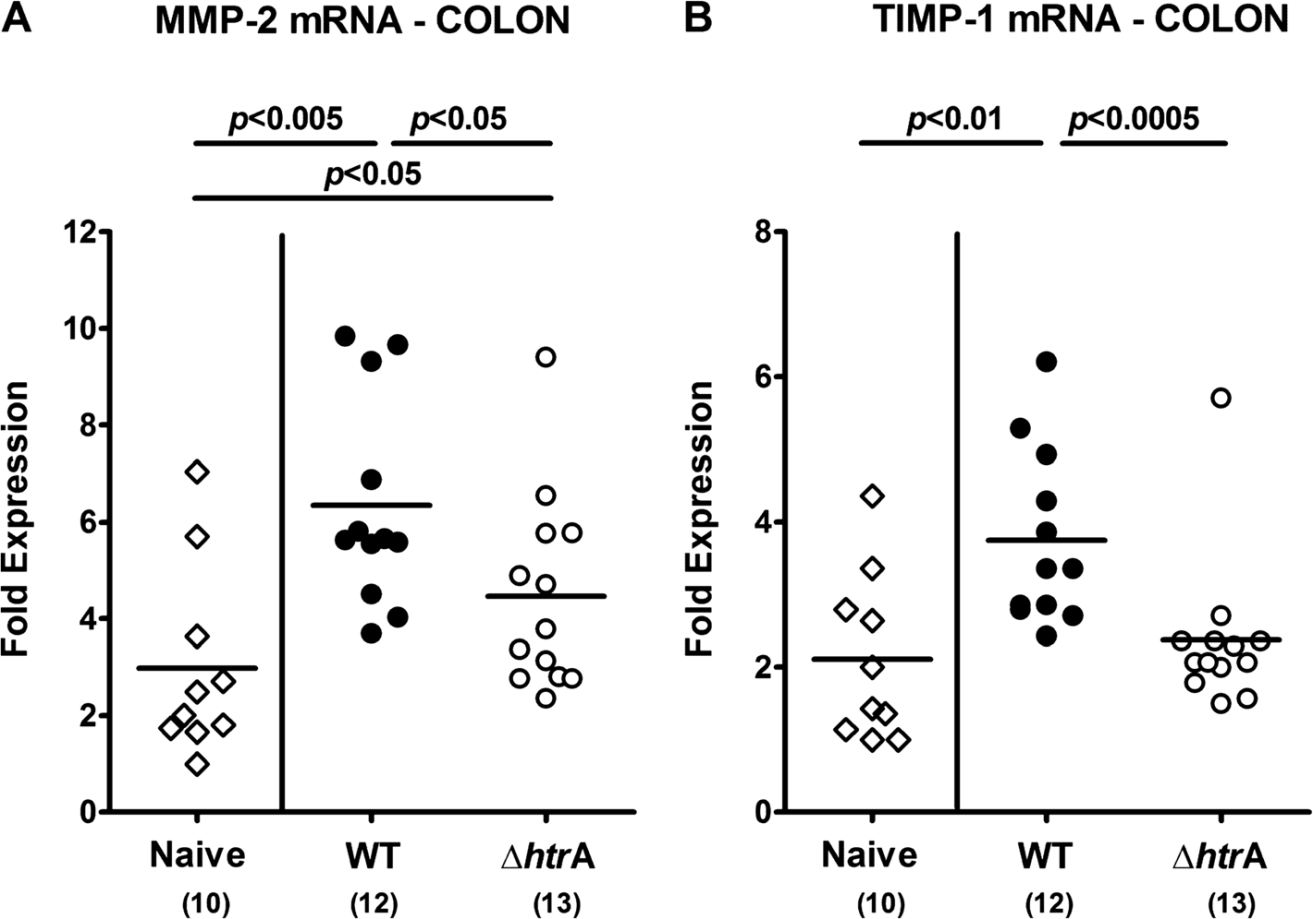
**Colonic matrixmetalloproteinase-2 expression following *C. jejuni *infection of infant mice.** Immediately after weaning, infant mice were perorally infected with *C. jejuni* NCTC11168 (WT, closed circles) or the mutant strain NCTC11168∆*htrA* (∆*htrA*, open circles). Uninfected animals (Naïve; open diamonds) served as negative controls. **(A)** MMP-2 and **(B)** TIMP-1 mRNA expression levels were determined in colonic *ex vivo* biopsies taken at day 7 p.i. by quantitative real-time PCR. Numbers of analyzed animals are given in parentheses. Means (black bars) and levels of significance (*P*-values) determined by the Mann–Whitney-U test are indicated. Data shown were pooled from three independent experiments.

Given that the matrix-degrading endopeptidase matrixmetalloproteinase (MMP)-2 is upregulated during intestinal immunopathology in mice and men [32-36], we next assessed colonic expression levels of MMP-2 and its endogenous inhibitor, the tissue inhibitor of matrixmetalloproteinase (TIMP)-1. Seven days following *C. jejuni* infection, MMP-2 mRNA expression levels increased multi-fold, but less distinctly in colons of ∆*htrA* as compared to parental strain infected mice (p < 0.05; Figure 5A). Furthermore, colonic TIMP-1 mRNA expression was up-regulated 7 days following parental but not ∆*htrA* strain infection (p < 0.01; Figure 5B). Taken together, absence of the *htrA* gene is associated with less distinct *C. jejuni* induced apoptosis and inflammation in the intestinal tract.

### *C. jejuni* HtrA plays a crucial role in the induction of extra-intestinal immune responses

**Figure 6:**
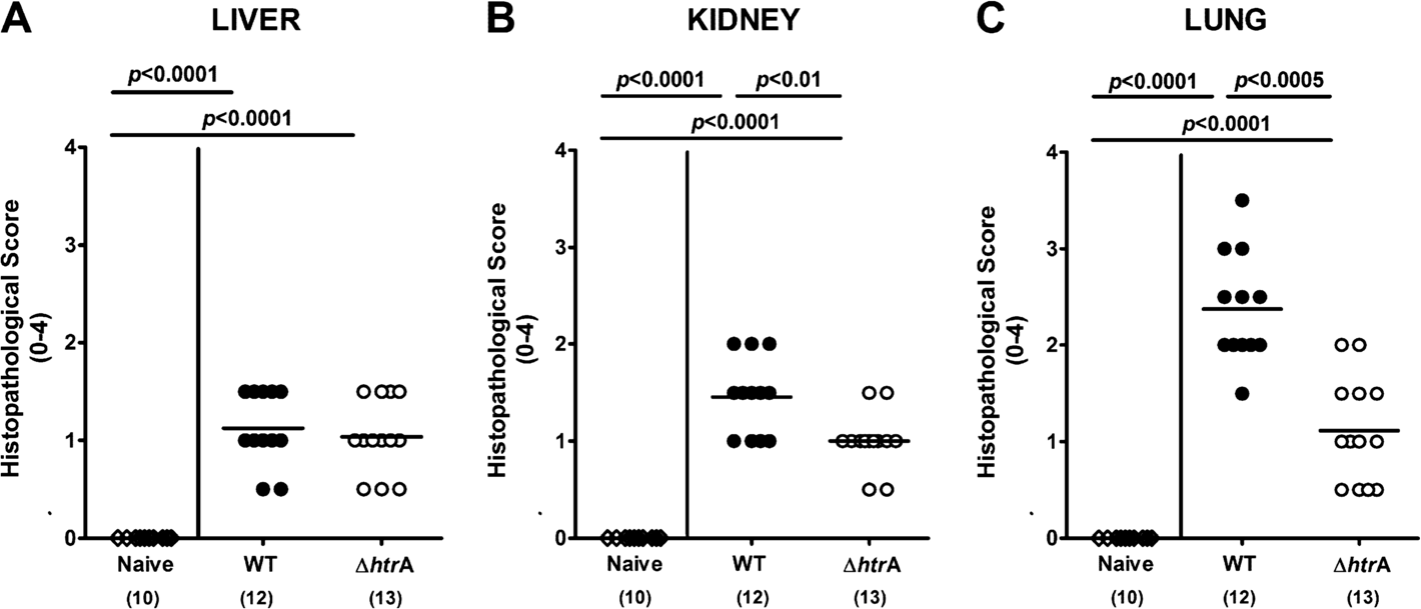
**Role of HtrA in extra-intestinal histopathological sequelae following *C. jejuni *infection of infant mice.** Immediately after weaning, infant mice were perorally infected with *C. jejuni* NCTC11168 (WT, closed circles) or the mutant strain NCTC11168∆*htrA* (∆*htrA*, open circles). Uninfected animals (Naïve; open diamonds) served as negative controls. Seven days following *C. jejuni* infection extra-intestinal immunopathological sequelae were assessed in H&E stained paraffin sections taken from **(A)** liver, **(B)** kidneys, and **(C)** lungs by applying respective standardized histopathological scoring systems. Means (black bars), levels of significance (*P*-values) determined by the Mann–Whitney-U test and numbers of analyzed animals (in parentheses) are indicated. Data shown were pooled from three independent experiments.

We have recently shown that asymptomatic longterm *C. jejuni* carrying mice displayed inflammatory immune responses in extra-intestinal compartments such as liver, kidneys and lungs [30]. We here investigated extra-intestinal *C. jejuni* induced sequelae as early as 7 days p.i. To address this we quantified inflammatory changes in H&E stained paraffin sections of liver, kidneys and lungs applying respective standardized histopathological scores. Remarkably, 7 days following *C. jejuni* infection with either strain mild to moderate histopathological changes could be observed in extra-intestinal organs that were exclusively lacking viable *C. jejuni* (Figure 6). Whereas only subtle inflammatory infiltrates could be observed in livers of infected infant mice irrespective of the *C. jejuni* strain (Figure 6A), histopathological scores for kidneys and lungs were lower in ∆*htrA* mutant as compared to parental strain infected animals at day 7 p.i. (Figure 6B, C), indicative for less distinct inflammatory disease in the respective extra-intestinal organs due to *htrA* deficiency of the *C. jejuni* strain.

Taken together, upon ∆*htrA* strain infection of infant mice large intestinal pro-inflammatory immune responses were ameliorated whereas compensatory regenerative/proliferating properties of the epithelium were preserved. Remarkably, *C. jejuni* induced inflammatory sequelae in extra-intestinal organs such as liver, kidneys and lungs could be observed as early as 7 days p.i., whereas extra-intestinal responses were less pronounced in the latter two compartments due to *htrA* deficiency.

## Discussion

We have recently shown *in vitro* that the chaperone and serine protease HtrA secreted by *C. jejuni* exerts a novel pathogenicity factor that is involved in bacterial invasion and transmigration across epithelial cells by cleaving E-cadherin and opening cell-to-cell junctions [20-23]. In the *in vivo* study presented here we investigated the impact of the *htrA* gene in pathogen-host-interaction and induction of immunopathology upon *C. jejuni* infection. To address this, conventionally colonized infant mice were infected either with the *C. jejuni* knockout mutant NCTC11168∆*htrA* or its syngenic parental WT strain at the age of 3 weeks immediatedly after weaning. Even though only a subset of mice harboured the respective strain in the intestinal tract, about one third of infected mice suffered from bloody diarrhea. In a previous infection study with another *C. jejuni* strain (B2), having highly efficient colonizing properties, virtually all infant mice harboured the pathogen at day 7 p.i., whereas up to 90% of mice displayed bloody diarrhea [30]. However, in our experiments with parental strain NCTC11168, but not ∆*htrA* mutant infected infant mice exhibited multi-fold increased numbers of colonic apoptotic cells at day 7 p.i. as compared to naïve controls. Conversely, the number of proliferating cells was significantly increased in ∆*htrA* but not parental strain infected mice indicative for up-regulated regenerative properties of intestinal epithelial cells thereby counteracting *C. jejuni* induced tissue damage. Less pronounced intestinal immunopathology due to the absence of HtrA was further underlined by lower expression levels of colonic pro-inflammatory cytokines such as TNF-α and IFN-γ, which have been shown to be key cytokines mediating *C. jejuni* induced immunopathology in murine infection models with different clinical severity [24,25]. Interestingly, less distinct intestinal immunopathology was accompanied by lower colonic expression levels of the matrix-degrading enzyme MMP-2 and its endogenous inhibitor TIMP-1 seven days following ∆*htrA* as compared to the parental strain infection. These MMP expression data are in good agreement with previous studies demonstrating that MMP-2 is up-regulated in acute and chronic small as well as large intestinal inflammation in mice and men [32,33,35-38]. For the first time we have now presented evidence that MMP-2 might also play an important role in mediating *C. jejuni*-induced disease, which is currently further unravelled in ongoing studies.

Surprisingly, rather mild to moderate histopathological sequelae of *C. jejuni* infection could be detected as early as one week in extra-intestinal organs such as liver, kidneys and lungs. All organ samples were free of viable *C. jejuni* as shown by negative cultures. In our previous study, *C. jejuni* B2 strain infected infant mice exhibited histopathological changes in the respective organs more than 100 days p.i. [30] with inflammatory foci consisting mainly of accumulated CD3-positive T cells [31]. Strikingly, in the present study, extra-intestinal histopathological changes in kidneys and lungs were less distinct one week following ∆*htrA* as compared to parental strain infection. Hence, absence of the HtrA protein is not only associated with less pronounced intestinal but also extra-intestinal inflammation.

In humans, only very few cases of pathogen-associated disease manifestations affecting liver, lungs, heart or spleen have been reported in severely immuno-compromized patients with *C. jejuni* bacteremia [39-41]. Fauchere and coworkers showed in isolator-raised germfree mice that *C. jejuni* was cleared from extra-intestinal compartments such as liver and spleen and the circulation within 24 hours following infection most likely due to non-specific bactericidal factors such as phagocytes and complement [42]. Histopathological changes within extra-intestinal organs, however, were not investigated [42]. In the context with our previous observation that CD3-positive cells accumulate at extra-intestinal locations, it is tempting to speculate that potentially pro-inflammatory immune cell populations might be attracted to the extra-intestinal compartments very early following infection before the subsequent clearing of the pathogen. These immune cells might then further reside in the respective organs and explain the sterile inflammatory responses in extra-intestinal tissue sites observed 7 days p.i. as well as in asymptomatic long-term *C. jejuni* carriers more than 100 days p.i. [30,31].

## Conclusions

Our *in vivo* study using the infant mouse infection model provides clear evidence for the importance of HtrA as a new virulence factor mediating *C. jejuni* induced intestinal as well as extra-intestinal immune responses. Thus, we describe here the first known *C. jejuni* mutant with very high motility [21], but having very low potential to trigger intestinal inflammation and bloody diarrhea as compared to WT bacteria. Future studies will further elucidate the underlying molecular mechanisms of *C. jejuni*-host-interactions.

## Materials and methods

### Ethics statement

All animal experiments were conducted according to the European Guidelines for animal welfare (2010/63/EU) with approval of the commission for animal experiments headed by the “Landesamt für Gesundheit und Soziales” (LaGeSo, Berlin, Germany; registration numbers G0123/12). Animal welfare was monitored twice daily by assessment of clinical conditions.

### Mice and *C. jejuni* infection

All mice were bred and maintained under specific pathogen-free (SPF) conditions in the facilities of the “Forschungseinrichtung für Experimentelle Medizin” (FEM, Charité - Universitätsmedizin, Berlin, Germany). Immediately after weaning, female 3-weeks-old C57BL/6 mice were infected orally with approximately 10^9^ viable CFU of the *C. jejuni* parental WT strain NCTC11168 or the isogenic mutant strain NCTC11168∆*htrA* lacking the *htrA* gene [21,22] by gavage in a total volume of 0.3 mL PBS on two consecutive days (day 0 and day 1).

### Clinical signs of *C. jejuni* infection, bloody feces

To assess clinical signs of *C. jejuni* induced infection, the occurrence of blood in fecal samples was determined applying a standardized score (0 points: no blood; 2 points: microscopic detection of blood by the Guajac method using Haemoccult, Beckman Coulter/PCD, Krefeld, Germany; 4 points: overt blood visible) [25,43].

### Sampling procedures and histopathology

Mice were sacrificed by isofluran treatment (Abbott, Germany). Tissue samples from liver, kidneys, lungs, and intestinal tract (duodenum, ileum, colon) were removed under sterile conditions. Intestinal samples from each mouse were collected in parallel for histopathological, immunohistochemical, microbiological, and immunological analyses. Immunohistopathological changes were determined in samples derived from colon, liver, kidneys and lungs that were immediately fixed in 5% formalin and embedded in paraffin. Sections (5 μm) were stained with H&E, examined by light microscopy (magnification 100× and 400×) and histopathological changes quantitatively assessed by two independent double-blinded investigators applying respective histopathological scoring systems. In brief:

**Colonic histopathology** (max. 4 points; according to [44]): 0: no inflammation; 1: single isolated cell infiltrates within the mucosa, no epithelial hyperplasia; 2: mild scattered to diffuse cell infiltrates within the mucosa and submucosa; mild epithelial hyperplasia; starting loss of goblet cells; 3: cell infiltrates within mucosa, submucosa, and sometimes transmural; epithelial hyperplasia; loss of goblet cells; 4: cell infiltrates within mucosa, submucosa, and transmural; severe inflammation; loss of goblet cells, loss of crypts; ulcerations; severe epithelial hyperplasia.

**Hepatic histopathology** (max. 9 points; modified Ishak score [45]): Lobular inflammation: 0: normal; 1: minimal inflammation (few inflammatory infiltrates); 2: mild inflammation (increased inflammatory cells, but less pyknotic necrosis); 3: moderate inflammation (marked increase in inflammatory cells and lots of pyknotic necroses); 4: severe inflammation (necrosis); 5: severe inflammation (plus bridging necroses).

Portal inflammation: 0: normal; 1: mild inflammation (<1/3 of portal tracts); 2: moderate inflammation (ca. 1/2 of portal tracts); 3: severe inflammation (>2/3 of portal tracts); 4: severe inflammation (plus portal inflammation disperse into parenchyma).

**Renal histopathology** (max. 4 points; according to [46]):

0: normal glomerulus; 1: focal and mild hypercellularity (normal = 3 per segment); 2: multifocal and moderate hypercellularity with capillary dilatation and mild hyalinosis; 3: diffuse hypercellularity (>50% of the tuft) and capillary aneurysm; 4: extensive sclerosis/crescents, tuft obliteration, collapse.

**Pulmonal histopathology** (max. 4 points, modified according to [47]):

0: no inflammation; 1: perivascular cuff of inflammatory cells; 2: mild inflammation, extending throughout <25% of the lung; 3: moderate inflammation covering 25-50% of the lung; 4: severe inflammation involving >50% of the lung.

### Immunohistochemistry

*In situ* immunohistochemical analyses of 5 μm thin colonic paraffin sections were performed as described previously [24,25,30,31,48]. Primary antibodies against cleaved caspase-3 (Asp175, Cell Signaling, USA, 1:200), Ki67 (TEC3, Dako, Denmark, 1:100), CD3 (M-20, Santa Cruz, 1:1000), and Foxp3 (FJK-16 s, eBioscience, 1:100) were used. For each animal the average number of positively stained cells within at least six high power fields (HPF, 0.287 mm^2^; 400× magnification) was determined microscopically by two independent double-blinded investigators.

### Quantitative analysis of *C. jejuni*

At time of necropsy (day 7 p.i.) live *C. jejuni* were detected in luminal samples derived from the duodenum, ileum or colon dissolved in sterile PBS by culture as described earlier [24,31]. In brief, serial dilutions of fecal samples were streaked out on karmali agar (Oxoid, Wesel, Germany) and incubated in a microaerobic atmosphere at 37°C for at least 48 hours. The respective weights of luminal fecal samples were determined by the difference of the sample weights before and after asservation.

### Cytokine detection in colonic *ex vivo* biopsies

Colonic biopsies were cut longitudinally and washed in PBS. Strips of approximately 1 cm^2^ colon were placed in 24-flat-bottom well culture plates (Nunc, Wiesbaden, Germany) containing 500 μL serum-free RPMI 1640 medium supplemented with penicillin (100 U/ mL) and streptomycin (100 μg/ mL; PAA Laboratories). After 18 h at 37°C supernatants were tested for TNF-α by ELISA (BD Biosciences).

### Real-time PCR analysis

RNA was isolated from colonic tissues using the RNeasy Mini Kit (Qiagen). mRNA was reversed transcribed and analysed in triplicate assays by TaqMan PCR using a sequence detection system (ABI Prism 7700; Applied Biosystems) as described previously [35,49]. For detection of murine IFN-γ, MMP-2 and TIMP-1 assays including double-fluorescent probes in combination with assays for the mouse housekeeping gene hypoxanthine phosphoribosyltransferase (HPRT) were purchased from Applied Biosystems). Expression levels were calculated relative to the HPRT expression.

### Antibodies and Western blotting

*C. jejuni* cell pellets were lysed and proteins were separated by SDS-PAGE [50,51]. Coomassie blue staining was done as described [52]. The polyclonal rabbit α-HtrA antibody was raised against a conserved peptide corresponding to amino acid (aa) residues 288–301: C-QGDTKKAYKNQEGA. The peptide was conjugated to *Limulus polyphemus* haemocyanin carrier protein, and two rabbits each were immunized by Biogenes GmbH (Berlin, Germany) using standard protocols [53]. The resulting antiserum was affinity-purified and the specificity against the proteins in *C. jejuni* was confirmed by Western blotting [54,55]. Horseradish peroxidase-conjugated anti-rabbit polyvalent sheep immunoglobulin was used as secondary antibody (DAKO Denmark A/S, DK-2600 Glostrup, Denmark). Blots were developed with ECL Plus Western blot reagents (GE Healthcare, UK limited Amersham Place, UK) as described [56,57].

### Statistical analysis

Mean values, medians, and levels of significance were determined using Mann–Whitney-U test. Two-sided probability (*P*) values ≤ 0.05 were considered significant. All experiments were repeated at least twice.

## Competing interests

The authors have declared that no competing interests exist.

## Authors’ contributions

Conceived and designed the experiments: MMH AF MA SB SB. Performed the experiments: MMH AF MA UG. Analyzed the data: MMH AF MA AAK MB NT SB SB. Contributed reagents/ materials/ analysis tolls: UBG MB NT AAK. Wrote the paper: MMH, SB, SB. All authors read and approved the final manuscript.
